# Sun Protection and Sunburn in Children Aged 1–10 Years in Germany: Prevalence and Determinants

**DOI:** 10.3390/children8080668

**Published:** 2021-07-31

**Authors:** Tatiana Görig, Corinna Södel, Annette B. Pfahlberg, Olaf Gefeller, Eckhard W. Breitbart, Katharina Diehl

**Affiliations:** 1 Mannheim Institute of Public Health, Social and Preventive Medicine, Mannheim Medical Faculty, Heidelberg University, 68167 Mannheim, Germany; Tatiana.Goerig@medma.uni-heidelberg.de (T.G.); Corinna.Soedel@medma.uni-heidelberg.de (C.S.); 2 Department of Medical Informatics, Biometry and Epidemiology, Friedrich-Alexander-University of Erlangen-Nuremberg, 91054 Erlangen, Germany; Annette.Pfahlberg@fau.de (A.B.P.); Olaf.Gefeller@fau.de (O.G.); 3 Association of Dermatological Prevention, 20457 Hamburg, Germany; info@professor-breitbart.de

**Keywords:** sun protection, measures, sunburn, sunscreen, children, role model, Germany

## Abstract

Seeking shade, the use of textile sun protection and sunscreen, and protecting one’s eyes by wearing sunglasses are recommended sun protection measures in children. We aimed to quantify the use of these measures as well as the prevalence of sunburn in children aged 1 to 10 years in Germany and to identify their determinants. Data collected via telephone interviews in a nationwide sample of 554 parents or caregivers in family were analyzed. Use of sunscreen was the most common measure applied (77.8%), while sunglasses were least frequently used (12.5%). The prevalence of sunburn during the past year was 21.8%, and it was positively associated with children’s age. The use of sun protection measures was significantly associated with the age and skin color of the child, while characteristics and tanning behaviors of the caregivers only played a minor role. The use of sun protection measures was higher when caregivers perceived themselves as a role model (Odds Ratio (OR) = 4.33, *p* < 0.001). Our nationwide data show that there remains a need for the improved use of sun protection measures, especially in children aged 7 to 10 years. In educational material, parents should be encouraged to become positive role models for their children regarding sun protection.

## 1. Introduction

Today, skin cancers—including cutaneous melanoma and keratinocyte carcinomas—are the most common incident cancers worldwide [1]. Proper sun protection behaviors can help to reduce skin cancer morbidity and mortality [2]. Sun protection includes a variety of measures. In agreement with international organizations and health authorities, national health authorities in Germany recommend avoiding high exposure to ultraviolet (UV) radiation by avoiding direct sun during UV peak hours around midday and spending as much time as possible in the shade, wearing protective clothing and headgear, using sunscreen on uncovered skin areas, and protecting one’s eyes by wearing sunglasses [2,3,4].

In order to reduce the skin cancer incidence, sun protection should be started as early in life as possible [5]. Especially young children are in specific need of protection—as stated in the Convention on the Rights of the Child by the United Nations—and they seem to be more vulnerable for several reasons. First, due to the nature of their skin, UV radiation can penetrate particularly deeply into the skin and cause irreversible damage [6,7] that, however, is difficult to detect clinically during childhood. Second, several studies have identified childhood and early adolescence to be a period in life during which a relatively higher cumulative amount of UV radiation is acquired than during adulthood [6,8]. Third, high UV exposure during early life has been shown to be associated with higher nevus prevalence, which in turn is related to the risk of melanoma development in later life [6,9,10]. Fourth, especially young children cannot protect themselves and thus depend on their parents [11], who therefore play an important role for sun protection during childhood. Moreover, parents can act as a role model for their children not only with their attitudes towards sun protection but especially with their own sun protection behavior [12,13,14,15].

Previous studies exploring sun protective practices of children have mostly reported a positive association with younger age [11,16,17,18,19] and sensitive fair skin [18,19,20], while mixed results are found for parents’ age [15,16] and children’s sex [18,19,20]. Although studies on sun protective guidance for children exist, it is often difficult to draw conclusions for nationwide prevention campaigns that target parents because studies have often been conducted in kindergarten [16,21], preschool [20], or school settings [13]. In addition, many studies have been conducted outside Europe [13,14,15,17,18,22], which does not allow the drawing of conclusions for European countries due to the different latitude. Studies from Germany comprise regionally limited samples from Bavaria and therefore do not allow for a comprehensive description of use of sun protection measures in children in Germany [16,20,21,23].

By using a nationwide sample, we pursued a three-fold aim in this paper. First, we aimed to quantify the use of different sun protection measures and prevalence of sunburn in 1- to 10-year-old children in Germany. Second, we aimed to identify determinants associated with sun protection based on a variety of variables, including characteristics and behaviors of children and children’s caregivers within the household they lived (primarily parents). Third, we analyzed the association between children’s sun protection and the subjectively perceived role-model function of the interviewed caregivers. Our findings can inform future prevention measures and help to identify specific starting points to improve sun protection in children.

## 2. Materials and Methods

### 2.1. Study Setting

Cross-sectional data were collected within the sixth wave of the Germany-wide National Cancer Aid Monitoring [24]. From October to December 2020, 4000 individuals aged 16–65 years were interviewed in computer-assisted telephone interviews (response rate: 29.8%). Participants were selected via a two-stage random sampling procedure reported elsewhere [25]. All participants provided informed consent to participate in this study. The Ethics Committee of the Medical Faculty Mannheim of Heidelberg University approved the study design and sampling procedures (2017-662N-MA).

This manuscript is based on data provided by a sub-sample comprising 554 participants who reported living in the same household as at least one child aged between one and ten years. In the following, this group will be referred to as caregivers. We used the approach suggested in previous research [18] and did not ask whether the responders were parents of the children. It is likely that primarily parents answered these questions, although grandparents and foster parents may also have been targeted by these questions. In households with more than one child of this age, caregivers were asked to relate their responses to the oldest child in this age group, as suggested in previous studies [26,27].

### 2.2. Instrument and Measures

All questions and response categories included in this manuscript were intensively pretested for comprehensibility and difficulty in responding in fifteen cognitive interviews.

#### 2.2.1. Outcome Variables Used to Describe Sun Protection in Children

Caregivers provided proxy reports regarding the application of six recommended sun protection measures by answering the following questions: “How often does the child wear sunscreen on his/ her face?”; “How often does the child use sunscreen on other body areas exposed to the sun?”; “How often does the child wear a shirt with sleeves that cover the shoulders?”; “How often does the child wear a cap or hat?”; “How often does the child wear sunglasses?”; and “How often does the child stay in the shade or under an umbrella?” [27]. International organizations recommend the use of these sun protection measures for children [3,28]. For answering these questions, the participants were asked to think about how often these measures are used on a sunny summer day when outside for more than ten minutes.

These variables were used in three different ways in the following analysis. First, response categories were dichotomized (always, often vs. sometimes, rarely, never) to perform bi- and multivariate analyses for each individual measure. Second, to illustrate overall sun protection of the children, we calculated a score combining the six dichotomous variables ranging from 0 (=none of the measures is conducted always/often) to 6 (=all measures are conducted always/often) for bivariate analyses. Third, for multivariable analyses of the overall sun protection, a dummy variable with values 0 (=applying ≤ 3 protective measures always or often) and 1 (=applying ≥ 4 protective measures always or often) based on the median split was coded.

Subsequently, the prevalence of sunburn of the child was gathered from caregivers using the question “In the past 12 months, how many times did this child have a painful or red sunburn that lasted a day or more?”, as recommended by Glanz et al. [27]. The responses were dichotomized (never vs. once or more often) to perform bi- and multivariate analyses.

#### 2.2.2. Covariates

The following covariates were used in this study:

*Characteristics of the child:* The participants provided information on the sex (male vs. female), age (1–3 years, 4–6 years, 7–10 years), and skin color of the child (very fair or fair skin vs. medium skin vs. brown or very brown skin).

*Sociodemographic characteristics of the caregivers:* The participants provided information on their sex (male vs. female), age, immigrant background (yes vs. no), the highest level of school education (categorized as low (still at school, without school-leaving qualification or general school) vs. medium (secondary school) vs. high (high school graduate)), and employment status (unemployed vs. part-time employed vs. full-time employed). Further, we asked about the federal state in which the participants live. The participants’ area of residence based on federal states was subsequently categorized into northern, southern, western, and eastern geographic regions in Germany.

*Skin characteristics and tanning behaviors of the caregivers:* We ascertained information on the self-reported skin type of the study participants using Fitzpatrick’s classification [29]. Tanning bed use in the last 12 months (yes vs. no) was assessed. In addition, intentional outdoor tanning on different occasions was assessed using three questions: “How often do you go in the sun in order to get a tan during your holidays?”; “In summer, how often do you go in the sun in order to get a tan on the weekend?”; and “In summer, how often do you go in the sun in order to get a tan on a typical workday?” (very often, often, sometimes, rarely, never). For analyses, a dummy variable was used for contrasting participants who never tanned intentionally versus those who tanned on one, two, or three occasions.

*Role model:* Participants’ agreement about being a role model regarding sun protection for their children was assessed using the statement “I try to set a good example with my own sun protection” [18] (strongly disagree, rather disagree vs. rather agree, strongly agree).

### 2.3. Statistical Analyses

Besides descriptive statistics, we used chi-squared tests, Mann–Whitney-U tests, and Kruskal–Wallis H tests to identify bivariate associations between the characteristics of children and caregivers on the one hand and outcome variables (individual sun protection measures, overall sun protection, and sunburn) on the other hand. The subsequent multiple logistic regression models included variables that were significant in crude (bivariate) logistic regression models (*p* < 0.05; see online supplement, Tables S1–S3). Reported are odds ratios (OR) and the 95% confidence intervals (CI). All analyses were performed using SPSS version 25 (IBM Corporation, Armonk, NY, USA) with a predefined level of significance of *p* < 0.05.

## 3. Results

Slightly more participants’ reports were available for girls (53.1%), children aged 7–10 years (75.3%), and those with a (very) fair to medium skin color (87.2%; Table 1). The sample of participants was nearly equally divided by sex, and the mean age was 34.4 years (SD = 11.4). The majority of participants had no immigrant background (85.2%), medium or high school education (84.1%), and were employed full-time employed (58.4%; Table 1). Regarding skin characteristics and tanning behavior, 38.4% of the participants reported having skin type I or II, a minority had used tanning beds within the last 12 months (4.3%), and 6.3% never tanned intentionally outdoors. The majority perceived themselves as a role model for their children regarding sun protection (84.3%; Table 1).

**Figure 1:**
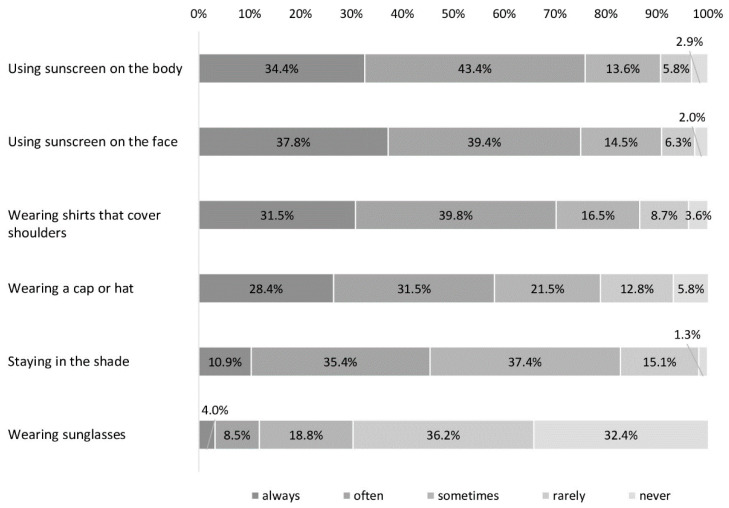
Use of sun protection measures in children aged between 1 and 10 years based on answers of 554 participants in wave 2020 of the National Cancer Aid Monitoring (NCAM).

Most of the participants stated that their child always or often uses sunscreen on their body (77.8%) and face (77.2%), and wears shirts covering the shoulders (71.3%; Figure 1). More than half of participants reported that their child wears a hat (59.9%). Staying in the shade and the use of sunglasses were less prevalent, with 46.3% and 12.5% indicating that they always or often used these measures, respectively (Figure 1).

Bivariate analyses showed that the use of almost all sun protection measures significantly differed depending on the age group and skin color of the child, with higher usage in children between 1 and 3 years and those with (very) fair skin (Table 2). We could not identify differences by sex.

Referring to the sociodemographic characteristics of caregivers, significant associations could be identified with the age of participants and using shirts covering the shoulders and sunglasses to protect their children (Table 2). However, no consistent age pattern could be identified for these items. Sunscreen use was higher (on the body: 79.6% vs. 67.1%, on the face: 79.0% vs. 67.1%) but use of sunglasses was lower (10.6% vs. 23.5%) for children whose caregivers had no immigrant background compared to those with an immigrant background. Applying sun-protective measures was not associated with the caregivers’ education level, employment status, or area of residence (Table 2).

In multivariate analyses, the use of all sun protection measures was lower in older children (except sunscreen use on the face and wearing sunglasses, Table 3). A significantly lower likelihood of sunscreen use on the body and face as well as wearing a hat could be identified in children with darker skin (Table 3). Significant associations with sociodemographic characteristics of the caregiver that were identified in preceding bivariate logistic regressions (see online supplement, Table S1) were not statistically significant in multiple logistic regressions. None of the skin and tanning characteristics of the caregivers were significant. Higher odds of sunscreen (e.g., OR = 3.64, *p* < 0.001 for using sunscreen on the body) and hat use (OR = 2.69, *p* < 0.001) as well as seeking shade (OR = 2.31, *p* = 0.002) could be identified in participants perceiving themselves as a role model for sun protection (Table 3).

When looking at the overall sun protection of the children, multivariate analyses showed lower odds for the use of ≥4 sun protective measures in older children (OR_7–10 years_ = 0.11, *p* < 0.001; Table 4). Children with (very) fair skin were more likely to use ≥4 sun protective measures (compared with those with (very) brown skin, *p* = 0.005). Caregivers with skin type I were significantly more likely to use ≥4 sun protective measures for their children than those with skin type II (*p* = 0.040). Participants who perceived themselves as a role model for sun protection were more likely to use of ≥4 sun protective measures in their children (OR = 4.33, *p* < 0.001; Table 4).

Overall, 21.8% of caregivers reported that their child had experienced at least one sunburn during the past 12 months. Sunburn was associated with increasing children’s age (OR_7–10 years_ = 4.81, *p* = 0.034; Table 5). Caregivers with a medium and high education level were less likely to report sunburn in their child (OR = 0.45, *p* = 0.022 and OR = 0.51, *p* = 0.022, respectively).

## 4. Discussion

Our nationwide survey in Germany showed that sunscreen use and wearing shirts that cover the shoulders are the most common sun protection measures in children aged 1 to 10 years, while seeking shade and the use of sunglasses are less prevalent. One in five caregivers reported their child sunburned at least once during the past 12 months. We found that caregivers’ sociodemographic characteristics and tanning behaviors only play a minor role in terms of sun protection of the child, since most associations were only significant in bivariate logistic regression models. By contrast, the child’s age and skin color are more important. This applies to the use of single measures as well as an overall measure combining the six recommended sun protection measures. In addition, the use of sun protection measures was higher when caregivers perceived themselves as role models.

Our findings on the use of individual sun protection measures are comparable with previous international research [17,18,20,21,30]. In these studies, the prevalence of sunscreen use ranged between 58% [18] and 89% [30], wearing protective clothes ranged between 75% [17] and 92% [30], wearing headgear ranged between 29% [17] and 96% [30], seeking shade ranged between 7% [21] and 69% [20], and the use of sunglasses ranged between 8% [17] and 63% [30]. However, since no standardized instrument for assessing sun protective behaviors has been established to date, considerable methodological differences should be kept in mind when comparing our findings with those of previous studies (e.g., differing definition of protection behaviors, variation in response categories). Nevertheless, comparable with previous research, our study shows a low use of sunglasses compared with other measures. This suggests that sunglasses have not yet been established as an important sun protective measure for children, but rather may be considered as a fashion accessory. It is therefore crucial to increase parents’ awareness of the importance of sunglasses to prevent eye damages in children caused by UV radiation.

In accordance with previous research, our study confirms that younger children are more likely to be better protected from the sun [11,16,17,18,21]. No significant associations could be identified for the children’s sex, with previous studies also providing inconclusive results in this regard. While some protection measures seem to be more prevalent in girls (e.g., shade, sunglasses), higher usage of headgears and protective clothes was found for boys [18,20]. 

We found that children with fairer skin are more likely to be protected from the sun, which is in line with studies from Australia [18] and Germany [20]. This finding may appear plausible upon first glance, given that darker skin is less UV sensitive and supposed to need less protection than fair skin. However, recent research shows that UV-induced damage also occurs in skin types IV–V, emphasizing that those with darker skin types also need sun protection [31]. This applies to an even greater extent to young children due to their age-related skin characteristics. Thus, it is important to focus on this issue in educational materials for parents.

Similar to previous international studies, we could not identify any association between caregivers’ sociodemographic characteristics as well as tanning behaviors and children’s sun protection [14,22]. No conclusive results regarding these characteristics were reported in another study from Germany [20]. Obviously, parental knowledge, attitudes, and social norms play a more central role for sun protection of children than sociodemographics [17,32] and tanning behaviors of the caregivers. In our study, we explored the association between children’s sun protection and the subjectively perceived role model status of caregivers for their children’s sun protection. We found that those who indicated setting a good example for their children regarding their own sun protection behavior were more likely to indicate that their child uses sun protection measures. This underlines that it is crucial to strengthen parental role modeling in information and education materials, as it seems to be an important aspect for promoting sun protection behaviors in children.

The prevalence of sunburn in our sample (21.8%) indicates that there is need for further improvement of children’s sun protection in Germany. Especially in older children—who sometimes can already take care of sun protection on their own—parents should not make it the sole responsibility of the child and should take corrective action. Since sunscreen use was the most common protective measure in our sample, parents should also be certain that they and their children use it correctly. A previous study from our research group showed deficits in the sunscreen use in the adult population [33], which also can lead to the occurrence of sunburn. These aspects should also be focused in information materials for parents/guardians.

### Limitations

Some limitations of our study should be taken into account when interpreting the findings. First, given that we used caregivers’ proxy reports to describe sun protection and sunburns in children, caregivers may have overreported use of sun protection measures and underreported sunburns due to social desirability or recall bias. However, proxy reports have also been widely used earlier [18,26], and we used core items that were recommended in previous research [27]. Nonetheless, studies that aim to validate this self-reported information by assessing objective data are needed [34]. Second, we do not know whether the interviewed participant is one of the children’s parents in each case, and we refrained from asking for this sensitive information in our interviews. However, previous studies have adopted a similar approach in their nationwide surveys [18]. Furthermore, it can be assumed that adults living in one household with a child are relatively well informed about the child’s sun protection. Third, compared with previous studies from Germany [16,20], our analyses comprised a relatively small sub-sample of caregivers. However, in our study, we were able to survey a nationwide sample, whereas previous studies have focused on regional samples. Fourth, since our survey focused on the overall sun protection measures recommended for children, we were not able to examine the use of each measure in detail. Thus, our data does not provide information on whether sunscreen use was in line with recommendations (e.g., kind of product, SPF, timing of application). Our previous survey showed, however, that sunscreen use in adults often did not comply with recommendations regarding timing of (re-)application and amount of sunscreen applied to the skin [33]. Therefore, it is important to address these aspects of sunscreen use in future studies focusing on children.

## 5. Conclusions

Our nationwide data show that German children are relatively well protected from the sun. Nevertheless, there remains a need for sun protection improvement, especially in children between seven and ten years, since almost one in four children of this age was reported to experience sunburn, and we found a decrease in the use of sun protection measure with the increasing age of the children. Education and prevention should keep in mind the notion that children become more autonomous when they enter preschool and may be more likely to care for sun protection on their own. However, parents and caregivers should be careful to check whether sun protection measures are being used, such as the use of a sufficient amount of sunscreen. Here, pediatricians could play a key role during routine medical check-ups. In educational programs, parents should be encouraged to become positive role models for sun protection since this was the main determinant in our analyses.

## Figures and Tables

**Table 1:** Characteristics of study participants in the National Cancer Aid Monitoring (NCAM) in wave 2020 who reported living in the same household with at least one child aged between 1 and 10 years (caregivers) and children.

	*n*	%/Mean (SD)
Children		
Sex		
Male	258	46.7
Female	294	53.1
Age group		
1–3 years	34	6.1
4–6 years	103	18.6
7–10 years	417	75.3
Age in years	554	7.6 (2.1)
Skin color		
(Very) fair skin	210	38.0
Medium skin	272	49.2
(Very) brown skin	71	12.8
Caregivers		
Sociodemographic characteristics		
Sex		
Male	255	46.0
Female	299	54.0
Age group		
16–25 years	124	22.4
26–35 years	223	40.3
36–45 years	116	20.9
46–55 years	56	10.1
56–65 years	35	6.3
Age in years	548	34.4 (11.4)
Immigrant background		
No	472	85.2
Yes	82	14.8
School education		
Low	74	15.8
Medium	120	25.6
High	274	58.5
Employment status		
Unemployed	64	12.1
Part-time	155	29.4
Full-time	308	58.4
Area of residence		
North	93	16.8
South	179	32.3
West	185	33.4
East	97	17.5
Skin characteristics and tanning behaviors		
Skin type		
I	58	10.5
II	154	27.9
III–IV	270	48.9
V–VI	70	12.7
Current tanning bed use		
No	530	95.7
Yes	24	4.3
Intentional outdoor tanning		
never	35	6.3
On 1 occasion	62	11.2
On 2 occasions	105	19.0
On 3 occasions	352	63.5
Role model		
Rather disagree	86	15.7
Rather agree	462	84.3

Due to missing data, the sum of subgroups does not equal the total sample size of 554 for all items. SD = standard deviation.

**Table 2:** Determinants related to the use of sun protection measures based on answers of 554 participants in wave 2020 of the National Cancer Aid Monitoring (NCAM).

	Sunscreen on the Body		Sunscreen on the Face		Shirt That Covers Shoulders		Cap or Hat		Staying in the Shade		Sunglasses	
	%	*p*-Value	%	*p*-Value	%	*p*-Value	%	*p*-Value	%	*p*-Value	%	*p*-Value
Children												
Sex		0.259		0.822		0.613		0.829		0.626		0.138
Male	75.6		76.7		70.2		59.3		47.5		10.1	
Female	79.6		71.6		72.1		60.2		45.4		14.3	
Age group		0.002		0.015		0.002		<0.001		<0.001		0.133
1–3 years	94.1		88.2		85.3		91.2		85.3		23.5	
4–6 years	86.4		85.4		82.5		72.8		58.3		11.7	
7–10 years	74.3		74.3		67.3		54.1		40.1		11.8	
Skin color		<0.001		<0.001		0.189		<0.001		0.003		0.197
(Very) fair skin	88.1		88.6		75.2		67.6		53.8		15.7	
Medium skin	76.8		75.4		69.9		59.6		44.4		10.3	
(Very) brown skin	50.7		50.5		74.8		38.0		31.0		11.3	
Caregivers												
Sociodemographic characteristics												
Sex		0.198		0.855		0.898		0.028		0.543		0.821
Male	75.3		76.9		71.0		54.9		44.9		12.2	
Female	79.9		77.5		71.5		64.1		47.5		12.8	
Age group		0.435		0.199		0.024		0.296		0.146		0.036
16–25 years	82.3		82.3		82.3		64.5		54.8		13.0	
26–35 years	77.6		78.9		69.1		59.2		41.9		7.6	
36–45 years	76.5		74.8		68.7		54.8		43.5		19.1	
46–55 years	69.6		67.9		60.7		67.9		52.7		16.1	
56–65 years	80.0		71.4		71.4		51.4		42.9		14.3	
Immigrant background		0.012		0.018		0.242		0.215		0.235		0.001
No	79.6		79.0		72.2		60.9		47.3		10.6	
Yes	67.1		67.1		65.9		53.7		40.2		23.5	
School education		0.661		0.629		0.615		0.565		0.446		0.126
Low	74.3		73.0		75.7		62.2		41.9		18.9	
Medium	77.5		75.8		75.0		63.3		51.3		13.3	
High	79.2		78.1		71.2		58.0		47.3		10.3	
Employment status		0.287		0.187		0.524		0.463		0.701		0.533
Unemployed	89.8		68.3		69.8		65.1		50.8		8.1	
Part-time	78.1		76.8		74.2		61.9		44.5		13.5	
Full-time	78.9		78.9		69.2		57.8		46.4		12.3	
Area of residence		0.098		0.054		0.143		0.770		0.238		0.633
North	81.7		78.5		68.8		57.0		52.7		16.1	
South	76.0		76.5		77.1		62.6		43.0		12.8	
West	81.6		82.2		70.3		60.0		49.2		11.4	
East	69.8		67.7		64.6		57.3		35.7		10.4	
Skin characteristics and tanning behaviors												
Skin type		0.013		0.004		0.404		0.194		0.783		0.013
I	91.2		86.0		80.7		71.9		47.4		19.3	
II	79.2		83.8		70.1		61.0		46.4		10.4	
III-IV	77.0		75.2		70.4		57.4		44.6		9.3	
V-VI	67.1		64.3		68.6		55.7		51.4		21.4	
Current tanning bed use		0.007		0.026		0.612		0.486		0.964		0.207
No	76.7		76.4		71.5		59.5		46.3		12.9	
Yes	100.0		95.8		66.7		66.7		45.8		4.2	
Intentional outdoor tanning		0.382		0.596		0.330		0.641		0.066		0.627
never	74.3		74.3		71.4		65.7		50.0		14.3	
On 1 occasion	74.2		71.0		66.1		56.5		58.1		16.1	
On 2 occasions	83.8		79.0		78.1		63.8		37.1		14.3	
On 3 occasions	76.9		78.1		70.1		58.7		46.6		11.1	
Role model		<0.001		<0.001		0.276		<0.001		<0.001		0.314
Rather disagree	52.3		57.0		66.3		37.2		29.1		9.3	
Rather agree	83.1		81.6		72.1		64.1		49.8		13.2	

*p*-values based on chi-squared tests.

**Table 3:** Logistic regression analyses on sun protection measures based on answers of 554 participants in wave 2020 of the National Cancer Aid Monitoring (NCAM).

	Sunscreen on the Body	Sunscreen on the Face	Shirt That Covers Shoulders	Cap or Hat	Staying in the Shade	Sunglasses
	OR (95%-CI)	OR (95%-CI)	OR (95%-CI)	OR (95%-CI)	OR (95%-CI)	OR (95%-CI)
Children						
Age of child						
1–3 years	Ref.		Ref.	Ref.	Ref.	
4–6 years	0.51 (0.10–2.49)		0.84 (0.28–2.47)	0.25 (0.07–0.92)	0.26 (0.09–0.74)	
7–10 years	0.28 (0.06–1.25)		0.39 (0.15–1.03)	0.13 (0.04–0.44)	0.14 (0.05–0.36)	
Skin color						
(Very) fair skin	Ref.	Ref.		Ref.	Ref.	
Medium skin	0.45 (0.25–0.79)	0.41 (0.23–0.72)		0.82 (0.54–1.26)	0.78 (0.53–1.15)	
(Very) brown skin	0.19 (0.09–0.41)	0.18 (0.09–0.37)		0.44 (0.23–0.84)	0.53 (0.29–0.98)	
Caregivers						
Sociodemographic characteristics						
Sex						
Male				Ref.		
Female				1.33 (0.92–1.91)		
Age of caregiver						
16–25 years		Ref.	Ref.		Ref.	
26–35 years		0.84 (0.45–1.54)	0.53 (0.31–0.91)		0.64 (0.40–1.03)	
36–45 years		0.64 (0.32–1.25)	0.53 (0.29–0.99)		0.74 (0.43–1.27)	
46–55 years		0.50 (0.23–1.10)	0.36 (0.18–0.74)		1.07 (0.55–2.12)	
56–65 years		0.42 (0.17–1.07)	0.59 (0.24–1.41)		0.60 (0.27–1.35)	
Immigrant background						
No	Ref.	Ref.				Ref.
Yes	0.64 (0.35–1.15)	0.68 (0.38–1.22)				1.87 (0.91–3.84)
School education						
Low						Ref.
Medium						0.66 (0.29–1.49)
High						0.51 (0.25–1.04)
Skin characteristics and tanning behaviors						
Skin type						
I	Ref.	Ref.		Ref.		Ref.
II	0.32 (0.10–1.02)	0.84 (0.33–2.15)		0.68 (0.33–1.37)		0.86 (0.31–2.42)
III–IV	0.44 (0.14–1.34)	0.70 (0.29–1.72)		0.68 (0.34–1.38)		0.55 (0.20–1.47)
V–VI	0.39 (0.11–1.38)	0.61 (0.22–1.71)		0.74 (0.32–1.72)		1.41 (0.48–4.14)
Role model						
Rather disagree	Ref.	Ref.		Ref.	Ref.	
Rather agree	3.64 (2.13–6.22)	2.74 (1.60–4.72)		2.69 (1.61–4.49)	2.31 (1.35–3.93)	
*n*	546	546	553	546	546	465

OR, odds ratio; CI, confidence interval; Ref., reference category.

**Table 4:** Bivariate analyses and logistic regression on overall sun protection in children based on answers of 554 participants in wave 2020 of the National Cancer Aid Monitoring (NCAM).

	Sum Score of Protection Measures ^(1)^		Use of ≥ 4 Protection Measures ^(2)^		
			Bivariate Analysis		Multiple Logistic Regression
	Mean (SD)	*p*-Value	%	*p*-Value	OR (95%-CI)
Children					
Sex		0.494		0.484	
Male	2.87 (1.23)		50.7		
Female	2.94 (1.32)		53.4		
Age of child		<0.001		<0.001	
1–3 years	3.70 (1.94)		90.0		Ref.
4–6 years	3.33 (1.07)		65.8		0.20 (0.06–0.75)
7–10 years	2.75 (1.29)		45.9		0.11 (0.03–0.38)
Skin color		<0.001		<0.001	
(Very) fair skin	3.33 (1.10)		63.2		Ref.
Medium skin	2.85 (1.21)		50.9		0.74 (0.48–1.14)
(Very) brown skin	2.04 (1.44)		26.9		0.37 (0.19–0.74)
Caregivers					
Sociodemographic characteristics					
Sex		0.052		0.241	
Male	2.85 (1.26)		49.8		
Female	2.97 (1.29)		54.4		
Age of caregiver		0.001		0.002	
16–25 years	3.25 (1.15)		65.1		Ref.
26–35 years	2.83 (1.22)		47.9		0.50 (0.30–0.83)
36–45 years	2.70 (1.55)		44.0		0.54 (0.30–0.97)
46–55 years	2.80 (1.23)		54.7		0.69 (0.34–1.41)
56–65 years	2.82 (1.25)		46.7		0.41 (0.18–0.94)
Immigrant background		0.026		0.005	
No	2.96 (1.23)		54.6		
Yes	2.66 (1.46)		40.0		
School education		0.461		0.591	
Low	2.98 (1.37)		56.4		
Medium	2.92 (1.30)		55.4		
High	2.98 (1.25)		51.5		
Employment status		0.430		0.554	
Unemployed	2.70 (1.31)		45.7		
Part-time	2.90 (1.28)		51.7		
Full-time	2.98 (1.24)		52.8		
Area of residence		<0.001		0.002	
North	3.01 (1.25)		55.5		
South	3.06 (1.14)		53.4		
West	2.99 (1.24)		57.5		
East	2.42 (1.45)		36.4		
Skin characteristics and tanning behaviors					
Skin type		0.005		0.027	
I	3.49 (1.05)		68.7		Ref.
II	3.04 (1.12)		52.5		0.47 (0.23–0.97)
III–IV	2.89 (1.26)		50.6		0.57 (0.28–1.16)
V–VI	2.70 (1.51)		45.6		0.56 (0.24–1.32)
Current tanning bed use		0.304		0.864	
No	2.90 (1.29)		52.1		
Yes	3.17 (0.84)		53.8		
Intentional outdoor tanning		0.011		0.352	
never	3.08 (1.41)		59.5		
On 1 occasion	2.95 (1.32)		57.3		
On 2 occasions	3.23 (1.16)		55.4		
On 3 occasions	2.81 (1.28)		49.5		
Role model		<0.001		<0.001	
Rather disagree	2.16 (1.27)		24.3		Ref.
Rather agree	3.08 (1.26)		58.3		4.33 (2.45–7.67)
*n*					543

^(1)^ Sun protection sum score ranges from 0 to 6. *p*-values based on Kruskal–Wallis H and Mann–Whitney U tests ^(2)^ Dependent variable: applying ≥4 protective measures when staying outside on a sunny summer day for longer than 10 min based on median split. *p*-values based on chi-squared tests. The logistic regression model included only variables that were significant in crude logistic regression analysis (see online supplement, Table S2). SD = standard deviation; OR, odds ratio; CI, confidence interval; Ref., reference category.

**Table 5:** Bivariate analyses and multiple logistic regression addressing children’s sunburn (at least one sunburn in the child in the past 12 months) based on answers of 554 participants in wave 2020 of the National Cancer Aid Monitoring (NCAM).

	Bivariate Analysis		Multiple Logistic Regression
	%	*p*-Value	OR (95%-CI)
Children			
Sex		0.860	
Male	21.5		
Female	22.1		
Age of child		0.035	
1–3 years	5.9		Ref.
4–6 years	18.6		3.53 (0.76–16.29)
7–10 years	23.9		4.81 (1.12–20.59)
Skin color		0.462	
(Very) fair skin	24.4		
Medium skin	20.7		
(Very) brown skin	18.3		
Caregivers			
Sociodemographic characteristics			
Sex		0.080	
Male	25.1		
Female	18.9		
Age of caregiver		0.670	
16–25 years	22.6		
26–35 years	20.2		
36–45 years	21.7		
46–55 years	20.4		
56–65 years	31.4		
Immigrant background		0.967	
No	21.7		
Yes	22.0		
School education		0.038	
Low	32.9		Ref.
Medium	18.3		0.45 (0.23–0.89)
High	20.1		0.51 (0.29–0.91)
Employment status		0.570	
Unemployed	16.1		
Part-time	22.6		
Full-time	21.2		
Area of residence		0.220	
North	26.9		
South	22.5		
West	22.3		
East	14.6		
Skin characteristics and tanning behaviors			
Skin type		0.847	
I	22.8		
II	24.2		
III–IV	20.7		
V–VI	20.3		
Current tanning bed use		0.603	
No	22.0		
Yes	17.4		
Intentional outdoor tanning		0.903	
never	11.8		
On 1 occasion	12.9		
On 2 occasions	21.2		
On 3 occasions	24.5		
Role model		0.321	
Rather disagree	25.9		
Rather agree	21.0		
*n*			466

*p*-values based on chi-squared tests. The logistic regression model included only variables that were significant in crude logistic regression analysis (see online supplement, Table S3). OR, odds ratio; CI, confidence interval; Ref., reference category.

## Data Availability

The data presented in this study are available upon reasonable request from the corresponding author.

## Supplementary Materials

The following are available online at https://www.mdpi.com/article/10.3390/children8080668/s1, Supplement Table S1: Determinants related to the use of sun protection measures in individual logistic regressions; Supplement Table S2: Determinants related to the use of ≥4 sun protection measures in individual logistic regressions; Supplement Table S3: Determinants related to sunburn in children (at least one sunburn in the past 12 months).
