# Supplementary material for: Sun Protection and Sunburn in Children Aged 1–10 Years in Germany: Prevalence and Determinants
Source: Children (Basel). 2021 Jul 31;8(8):668. doi: 10.3390/children8080668 (PMC8394729; doi:10.3390/children8080668)
Supplement: Supplementary file 1 [file children-08-00668-s001.zip › children-1275459-supplementary.pdf]

Table S1. Determinants related to the use of sun protection measures in individual logistic regressions

	Sunscreen on the body		Sunscreen on the face		Shirt that covers shoulders		Cap or hat		Staying in the shade		Sunglasses	
	OR	[95%-CI]	OR	[95%-CI]	OR	[95%-CI]	OR	[95%-CI]	OR	[95%-CI]	OR	[95%-CI]
**Children**												
Sex												
Male	Ref.		Ref.		Ref.		Ref.		Ref.		Ref.	
Female	1.26	[0.84-1.88]	1.05	[0.70-1.56]	1.10	[0.76-1.59]	1.04	[0.74-1.46]	0.92	[0.66-1.29]	1.48	[0.88-2.49]
Age of child												
1-3 years	Ref.		Ref.		Ref.		Ref.		Ref.		Ref.	
4-6 years	0.40	[0.09-1.85]	0.78	[0.24-2.54]	0.81	[0.28-2.39]	0.26	[0.07-0.92]	0.24	[0.09-0.67]	0.43	[0.16-1.16]
7-10 years	0.18	[0.04-0.77]	0.39	[0.13-1.12]	0.36	[0.13-0.94]	0.11	[0.03-0.38]	0.12	[0.04-0.30]	0.44	[0.19-1.02]
Skin color												
(Very) fair skin	Ref.		Ref.		Ref.		Ref.		Ref.		Ref.	
Medium skin	0.45	[0.27-0.74]	0.40	[0.24-0.66]	0.76	[0.51-1.15]	0.71	[0.48-1.03]	0.69	[0.48-0.99]	0.62	[0.36-1.06]
(Very) brown skin	0.14	[0.07-0.26]	0.13	[0.07-0.25]	0.61	[0.34-1.08]	0.29	[0.17-0.51]	0.39	[0.22-0.68]	0.68	[0.30-1.55]
**Caregivers**												
**Sociodemographic characteristics**												
Sex												
Male	Ref.		Ref.		Ref.		Ref.		Ref.		Ref.	
Female	1.30	[0.87-1.94]	1.04	[0.70-1.55]	1.03	[0.71-1.48]	1.47	[1.04-2.06]	1.11	[0.79-1.55]	1.06	[0.64-1.76]
Age of caregiver												
16-25 years	Ref.		Ref.		Ref.		Ref.		Ref.		Ref.	
26-35 years	0.75	[0.43-1.30]	0.81	[0.46-1.42]	0.48	[0.28-0.83]	0.80	[0.51-1.26]	0.59	[0.38-0.93]	0.55	[0.27-1.14]
36-45 years	0.70	[0.37-1.32]	0.64	[0.34-1.19]	0.47	[0.26-0.87]	0.67	[0.40-1.12]	0.63	[0.38-1.06]	1.58	[0.79-3.19]
46-55 years	0.50	[0.24-1.03]	0.46	[0.22-0.94]	0.33	[0.16-0.68]	1.16	[0.59-2.27]	0.92	[0.49-1.74]	1.28	[0.53-3.11]
56-65 years	0.86	[0.33-2.23]	0.54	[0.23-1.28]	0.54	[0.23-1.28]	0.58	[0.27-1.24]	0.62	[0.29-1.32]	1.12	[0.38-3.29]
Immigrant background												
No	Ref.		Ref.		Ref.		Ref.		Ref.		Ref.	

Yes	0.52	[0.31-0.87]	0.54	[0.33-0.90]	0.74	[0.45-1.22]	0.74	[0.46-1.19]	0.75	[0.47-1.21]	2.58	[1.43-4.66]
School education												
Low	Ref.		Ref.		Ref.		Ref.		Ref.		Ref.	
Medium	1.19	[0.61-2.34]	1.16	[0.60-2.25]	0.96	[0.49-1.89]	1.05	[0.58-1.91]	1.46	[0.81-2.62]	0.66	[0.30-1.45]
High	1.32	[0.72-2.39]	1.32	[0.73-2.38]	0.73	[0.44-1.43]	0.84	[0.50-1.43]	1.24	[0.74-2.09]	0.49	[0.24-0.99]
Employment status												
Unemployed	Ref.		Ref.		Ref.		Ref.		Ref.		Ref.	
Part-time	1.54	[0.80-2.97]	1.54	[0.80-2.94]	1.24	[0.65-2.37]	0.87	[0.47-1.61]	0.78	[0.43-1.40]	1.79	[0.64-4.97]
Full-time	1.61	[0.88-2.95]	1.74	[0.96-3.16]	0.97	[0.54-1.75]	0.74	[0.41-1.29]	0.84	[0.49-1.44]	1.60	[0.61-4.25]
Area of residence												
North	Ref.		Ref.		Ref.		Ref.		Ref.		Ref.	
South	0.71	[0.38-1.33]	0.89	[0.49-1.63]	1.53	[0.87-2.67]	1.26	[0.76-2.10]	0.68	[0.41-1.12]	0.77	[0.38-1.55]
West	0.99	[0.52-1.89]	1.26	[0.68-2.35]	1.07	[0.62-1.84]	1.13	[0.68-1.88]	0.87	[0.53-1.43]	0.67	[0.33-1.37]
East	0.52	[0.26-1.02]	0.57	[0.30-1.11]	0.83	[0.45-1.52]	1.01	[0.57-1.80]	0.61	[0.34-1.09]	0.61	[0.26-1.42]
**Skin characteristics and tanning behaviors**												
Skin type												
I	Ref.		Ref.		Ref.		Ref.		Ref.		Ref.	
II	0.37	[0.14-0.99]	0.84	[0.36-1.99]	0.56	[0.27-1.18]	0.61	[0.32-1.19]	0.96	[0.52-1.77]	0.49	[0.21-1.12]
III-IV	0.32	[0.12-0.84]	0.50	[0.22-1.10]	0.57	[0.28-1.15]	0.53	[0.28-0.98]	0.89	[0.51-1.59]	0.43	[0.20-0.93]
V-VI	0.20	[0.07-0.56]	0.29	[0.12-0.72]	0.52	[0.23-1.20]	0.49	[0.23-1.04]	1.18	[0.58-2.37]	1.14	[0.48-2.73]
Current tanning bed use												
No	*	-	Ref.		Ref.		Ref.		Ref.		Ref.	
Yes	-	-	7.12	[0.95-53.23]	0.80	[0.34-1.91]	1.36	[0.57-3.23]	0.98	[0.43-2.23]	0.29	[0.04-2.21]
Intentional outdoor tanning												
never	Ref.		Ref.		Ref.		Ref.		Ref.		Ref.	
on 1 occasion	1.00	[0.39-2.57]	0.85	[0.33-2.16]	0.78	[0.32-1.93]	0.68	[0.29-1.60]	1.39	[0.60-3.21]	1.15	[0.36-3.70]
on 2 occasions	1.79	[0.72-4.49]	1.31	[0.54-3.19]	1.43	[0.60-3.39]	0.92	[0.41-2.05]	0.59	[0.27-1.29]	1.00	[0.34-2.98]
on 3 occasions	1.15	[0.52-2.56]	1.23	[0.55-2.74]	0.94	[0.44-2.02]	0.74	[0.36-1.54]	0.87	[0.43-1.76]	0.75	[0.28-2.05]
**Role model**												

rather disagree	Ref.		Ref.		Ref.		Ref.		Ref.		Ref.	
rather agree	4.49	[2.75-7.31]	3.35	[2.06-5.45]	1.31	[0.80-2.15]	3.01	[1.87-4.85]	2.42	[1.47-3.99]	1.49	[0.68-3.23]

n=554 individuals 16-65 years of age who participated in wave 2020 of the National Cancer Aid Monitoring (NCAM) and reported living in the same household as at least one child aged between 1 and 10 years

Ref.=Reference category, OR=Odds Ratio, CI=Confidence Interval

* Excluded in this analysis due to low case number

Table S2. Determinants related to the use of ≥ 4 sun protection measures in individual logistic regressions

	OR [95%-CI]
**Children**	
Sex	
Male	Ref.
Female	1.21 [0.87-1.70]
Age of child	
1-3 years	Ref.
4-6 years	0.20 [0.06-0.69]
7-10 years	0.09 [0.03-0.29]
Skin color	
(Very) fair skin	Ref.
Medium skin	0.64 [0.44-0.92]
(Very) brown skin	0.26 [0.15-0.46]
**Caregivers**	
**Sociodemographic characteristics**	
Sex	
Male	Ref.
Female	1.25 [0.90-1.76]
Age of caregiver	
16-25 years	Ref.
26-35 years	0.52 [0.33-0.82]
36-45 years	0.51 [0.30-0.86]
46-55 years	0.62 [0.33-1.19]
56-65 years	0.49 [0.23-1.05]
Immigrant background	
No	Ref.
Yes	0.64 [0.40-1.03]
School education	
Low	Ref.
Medium	1.03 [0.57-1.86]
High	0.80 [0.48-1.34]
Employment status	
Unemployed	Ref.
Part-time	1.18 [0.66-2.13]
Full-time	1.16 [0.67-1.99]
Area of residence	
North	Ref.
South	0.89 [0.54-1.48]
West	1.12 [0.68-1.85]
East	0.61 [0.34-1.09]
**Skin characteristics and tanning behaviors**	
Skin type	
I	Ref.
II	0.44 [0.23-0.85]
III-IV	0.42 [0.23-0.79]
V-VI	0.37 [0.18-0.78]
Current tanning bed use	
No	Ref.
Yes	1.76 [0.74-4.18]
Intentional outdoor tanning	

never	Ref.
on 1 occasion	0.91 [0.39-2.12]
on 2 occasions	0.86 [0.39-1.89]
on 3 occasions	0.77 [0.38-1.58]
**Role model**	
rather disagree	Ref.
rather agree	4.27 [2.54-7.18]

n=554 individuals 16-65 years of age who participated in wave 2020 of the National Cancer Aid Monitoring (NCAM) and reported living in the same household as at least one child aged between 1 and 10 years
Dependent variable: applying ≥ 4 protection measures when staying outside on a sunny summer day for longer than 10 minutes based on median split
OR = odds ratio; CI = confidence interval; Ref. = reference category

Table S3. Determinants related to sunburn in children (at least one sunburn in the past 12 months)

	OR [95%-CI]
**Children**	
Sex	
Male	Ref.
Female	1.03 [0.69-1.55]
Age of child	
1-3 years	Ref.
4-6 years	3.66 [0.81-16.63]
7-10 years	5.01 [1.18-21.29]
Skin color	
(Very) fair skin	Ref.
Medium skin	0.80 [0.52-1.24]
(Very) brown skin	0.69 [0.35-1.37]
**Caregivers**	
**Sociodemographic characteristics**	
Sex	
Male	Ref.
Female	0.69 [0.46-1.04]
Age of caregiver	
16-25 years	Ref.
26-35 years	0.87 [0.51-1.48]
36-45 years	0.95 [0.52-1.76]
46-55 years	0.88 [0.40-1.92]
56-65 years	1.57 [0.69-3.60]
Immigrant background	
No	Ref.
Yes	1.01 [0.57-1.78]
School education	
Low	Ref.
Medium	0.45 [0.23-0.89]
High	0.51 [0.29-0.91]
Employment status	
Unemployed	Ref.
Part-time	1.39 [0.67-2.89]
Full-time	1.51 [0.69-3.29]
Area of residence	
North	Ref.
South	0.78 [0.44-1.40]
West	0.78 [0.43-1.38]
East	0.46 [0.22-0.96]
**Skin characteristics and tanning behaviors**	
Skin type	
I	Ref.
II	1.08 [0.52-2.22]
III-IV	0.88 [0.44-1.75]
V-VI	0.86 [0.36-2.02]
Current tanning bed use	
No	Ref.
Yes	0.74 [0.24-2.24]
Intentional outdoor tanning	

never	Ref.
on 1 occasion	1.11 [0.30-3.99]
on 2 occasions	2.01 [0.64-6.32]
on 3 occasions	2.43 [0.83-7.01]
**Role model**	
rather disagree	Ref.
rather agree	0.76 [0.44-1.30]

n=554 individuals 16-65 years of age who participated in wave 2020 of the National Cancer Aid Monitoring (NCAM) and reported living in the same household as at least one child aged between 1 and 10 years
OR = odds ratio; CI = confidence interval; Ref. = reference category
